# Effect of amplitude and duration of impulsive pressure on endothelial permeability in *in vitro* fluid percussion trauma

**DOI:** 10.1186/1475-925X-13-44

**Published:** 2014-04-16

**Authors:** Hiromichi Nakadate, Koji Inuzuka, Suguru Akanuma, Akira Kakuta, Shigeru Aomura

**Affiliations:** 1 Graduate School of System Design, Tokyo Metropolitan University, Tokyo, Japan; 2 Advanced Course of Mechanical and Computer Systems Engineering, Tokyo National College of Technology, Tokyo, Japan

**Keywords:** Head impact, Impulsive pressure, Fluid percussion, Positive pressure, Negative pressure, Pressure amplitude, Pressure duration, Endothelial permeability, Transendothelial electrical resistance, Vascular endothelial cadherin

## Abstract

**Background:**

Intracranial pressure changes during head impact cause brain injuries such as vasogenic edema and cerebral contusion. However, the influence of impulsive pressure on endothelial function has not yet been fully studied *in vitro*. In this study, we developed a pressure loading device that produced positive and negative pressures by modifying an *in vitro* fluid percussion model and examined the effects of the amplitude and duration of the pressures on endothelial permeability.

**Methods:**

Human umbilical vein endothelial cells were subjected to three types of positive pressure (average amplitude/average duration of 352 kPa/23 ms, 73 kPa/27 ms, and 70 kPa/44 ms) and three types of negative pressure (−72 kPa/41 ms, −67 kPa/104 ms, and −91 kPa/108 ms), and the transendothelial electrical resistance (TEER) was measured between 15 min and 24 h after pressure loading for quantifying the formation of an integral monolayer of endothelial cells. After loading, vascular endothelial- (VE-) cadherin, an endothelium-specific cell-cell adhesion molecule involved in endothelial barrier function, was stained and observed using fluorescence microscopy.

**Results:**

The pressure loading device could produce positive pressure pulses with amplitudes of 53–1348 kPa and durations of 9–29.1 ms and negative pressure pulses with amplitudes of −52–−93 kPa and durations of 42.9–179.5 ms. The impulsive pressure reduced the TEER associated with the change in VE-cadherin localization. Additionally, TEER decreased considerably at 15 min and 6 h post-loading, with these changes being significant in positive pressure with larger amplitude and shorter duration and in all types of negative pressures compared to pre-loading.

**Conclusions:**

The changes in intracranial pressure during head impact impair endothelial barrier function by the disruption of the integrity of endothelial cell-cell junctions, and the degree of increase in endothelial permeability depends on the amplitude, duration, and direction (compressive and tensile) of the impulsive pressure.

## Background

Head impact causes a dynamic mechanical response such as a pressure gradient within the brain and shear deformation of the brain tissue, resulting in brain injury [1,2]. Animal studies of head impact have shown that the transient increase in intracranial pressure causes brain haemorrhage and cerebral concussion [3,4]. Typically, the pressure generated at the impact site (coup pressure) was positive and that generated on the side opposite to the impact site (contrecoup pressure) was negative in experiments using human cadavers [5-7]. We have demonstrated that positive and negative pressure fluctuations were generated at the impact site and the opposite side through a finite element simulation of nine real-world accident cases involving fatal cerebral contusion [8]. To date, various *in vitro* models have been developed for understanding the mechanical stimuli during an impact event and the subsequent responses of tissue and cells [9]. A fluid percussion model produced a transient pressure pulse during head impact more accurately [10,11]; however, the difference between positive and negative pressures has not been examined in *in vitro* models of trauma. Studies of experimental animals using a fluid percussion injury model and controlled cortical impact model have shown that cerebral vascular dysfunction such as morphologic damage to endothelial cells and increased permeability of blood-brain barrier (BBB) forms a physical barrier to filter blood-borne substances between the blood and the brain parenchyma [12]; however, the effects of pressure on cultured cells have been poorly studied relative to the effects of deformation, and few studies have focused on the endothelial cells of brain microvessels [11,13,14], which define one component of BBB, compared to central nervous system (CNS) cells such as neurons, astrocytes, and glial cells. In addition, the direct influence of impulsive pressure during head impact on the structure and function of endothelial cells *in vitro* has not yet been fully studied.

This study aims to better understand the contribution of brain mechanical responses to cerebral vascular injury resulting in vasogenic edema and cerebral contusion. Previously, we have demonstrated that transient positive and negative pressures increased the endothelial permeability to FITC-dextran [15]. In the present study, we examined the effects of the amplitude and duration of positive and negative pressures on the capillary-like structure and endothelial permeability using a pressure loading device developed by modifying an *in vitro* fluid percussion model.

## Methods

### Endothelial cell culture

Human umbilical vein endothelial cells (HUVEC) purchased from Lonza (Walkersville, MD, USA) were cultured in Endothelial Basal Medium-2 (EBM-2) supplemented with an EGM-2 SingleQuots containing recombinant human epidermal growth factor (rhEGF), heparin, hydrocortisone, recombinant human fibroblast growth factor-basic (rhFGF-B), ascorbic acid, recombinant human vascular endothelial growth factor (VEGF), recombinant long r insulin-like growth factor-1 (R^3^-IGF-1), gentamicin sulphate amphotericin-B (GA-1000), and foetal bovine serum (FBS) under conditions of 5% CO_2_ and 100% humidity at 37°C. HUVEC from passages 3–5 were seeded at 50×10^4^ cells on BD Matrigel matrix-coated 35-mm culture dishes (Asahi Technoglass, Tokyo, Japan) and were grown to tube formations within 24 h for evaluating the capillary-like structure. HUVEC from passages 3–5 were seeded at 4.4×10^5^ cells/well into cell culture inserts (6-well format, 0.4-μm pore size, BD, Franklin Lakes, NJ, USA) and were grown to confluent monolayers within 24 h for evaluating the endothelial cell function.

### Pressure loading device

**Figure 1:**
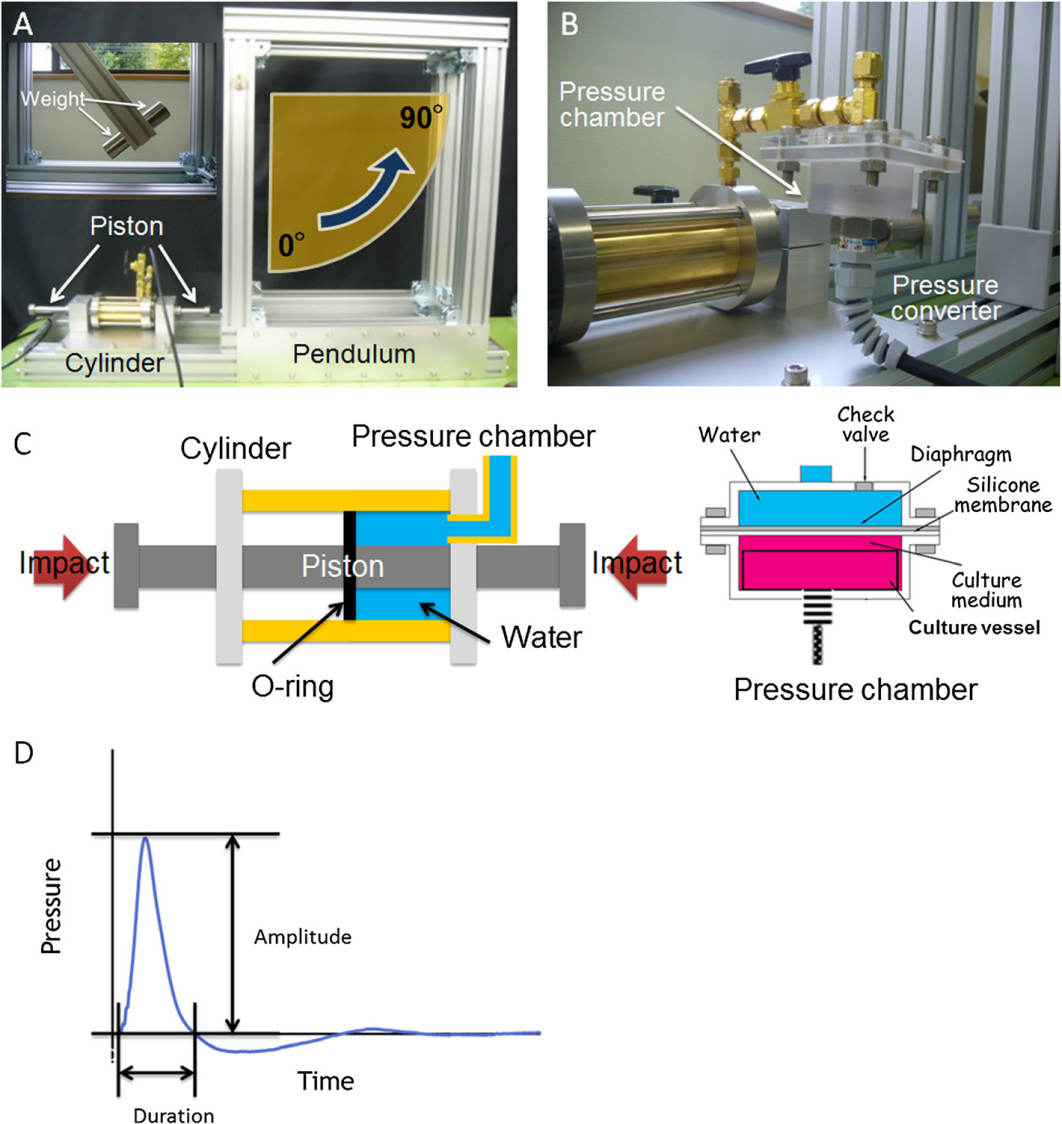
**Pressure loading device.** The pressure loading device consisted of a cylinder, a piston, and a pendulum impactor equipped with a weight **(A)**. A pressure chamber equipped with a pressure transducer is connected to the cylinder **(B)**. The compartment connected between the cylinder and the pressure chamber is filled with water. The lower part of the pressure chamber, which is divided by a silicone membrane, is filled with culture medium. Positive pressure or negative pressure is generated by striking the piston with the pendulum from the right or the left **(C)**. The peak pressure and time of an initial pulse in the generated pressure wave is referred to as the pressure amplitude and pressure duration **(D)**.

We employed the percussion of a pendulum, as described by Shepard et al. [10], on a pressure loading device consisting of a cylinder, a piston, a pendulum with a weight of 1 or 2 kg, a pressure chamber, and a pressure transducer (Figure 1A and B). The angle of the pendulum, having a radius of 500 mm and attached to an aluminium frame, can be adjusted from 0° to 90°. Figure 1C shows a cross-sectional diagram of the cylinder, piston, and pressure chamber. The pressure chamber is divided by a 0.5-mm silicone membrane. The upper stainless-steel compartment is filled with water and is connected to the cylinder. The lower polycarbonate compartment including the culture dish or the culture insert is filled with culture medium and connected to a pressure transducer with a working range of −98.07–2000 kPa (PGM-20KH, Kyowa, Tokyo). We designed and produced the cylinder and piston unit using brass and stainless-steel so as to produce both positive and negative pressures. The cylinder had a length and diameter of 100 mm and 35 mm, respectively. The piston’s corresponding dimensions were 410 mm and 16 mm. Two striking heads were attached at either end of the piston, and an O-ring was attached in the middle of the piston. One compartment, which is separated from the cylinder by the O-ring and is connected to the pressure chamber, is filled with water; therefore, the change in the striking direction made it possible to switch positive and negative pressure. The impulsive pressure shown in Figure 1D is generated by striking the piston with the weight attached to the pendulum. In the case of impact from the left, the water in the cylinder is compressed and positive pressure is generated; this pressure propagates to the pressure chamber. In the case of impact from the right, the water in the cylinder is stretched and negative pressure is generated.

### Measurement of transendothelial electrical resistance (TEER)

Following impulsive pressure loading, the cell culture insert was transferred from the pressure chamber into an EndOhm chamber (World Precision Instruments, Sarasota, FL, USA), and the electrical resistance across HUVEC monolayers was measured using an EVOM^2^ resistance meter (World Precision Instruments). The electrical resistance of blank inserts was subtracted from the results. In some experiments, HUVEC were incubated with a culture medium containing ethyleneglycotetraacetic acid (EGTA), a calcium chelator, with a final concentration of 2.5 mM at 37°C for 20 min. Fresh medium was then added after EGTA was removed, and the electrical resistance was measured without impulsive pressure loading. Lower TEER disrupts the endothelial integrity and in turn, the intercellular gap formation. Extracellular Ca^2+^ depletion by EGTA is followed by the loss of cell-cell adhesion proteins and induced intercellular gap formation [16].

### Immunofluorescence staining

After impulsive pressure loading and incubation with EGTA, HUVEC were rinsed with Hanks’ Balanced Salt Solution (HBSS) without Ca^2+^ and Mg^2+^ and fixed with 4% paraformaldehyde in phosphate buffered saline (PBS) for 10 min at 4°C; vascular endothelial- (VE-) cadherin, an endothelium-specific cell-cell adhesion molecule involved in the endothelial barrier function [17], was stained with 1 μg/mL rabbit anti-human VE-cadherin polyclonal antibody (BMS158, Bender MedSystems, CA, USA) as the primary antibody overnight at room temperature and with goat anti-rabbit IgG fluorescein conjugate (sc-2021, Santa Cruz Biotechnology, TX, USA) at a dilution of 1:400 as the secondary antibody overnight at room temperature. After HUVEC were washed with PBS, fluorescence images were observed using a fluorescence microscope (IX71, Olympus, Japan) equipped with a cooled CCD camera (Penguin 600CL, Pixera, Japan).

### Statistical analysis

The amplitude and duration of pressure pulses are expressed as the mean ± deviation (SD) of 3 independent experiments. The density of capillary-like network and TEER are expressed as the mean ± standard error of the mean (SEM) of 3–6 independent experiments. The means were compared by Steel’s multiple comparison test. A *p* value of less than 0.05 was considered significant.

## Results

### Outputs of pressure loading device

**Figure 2:**
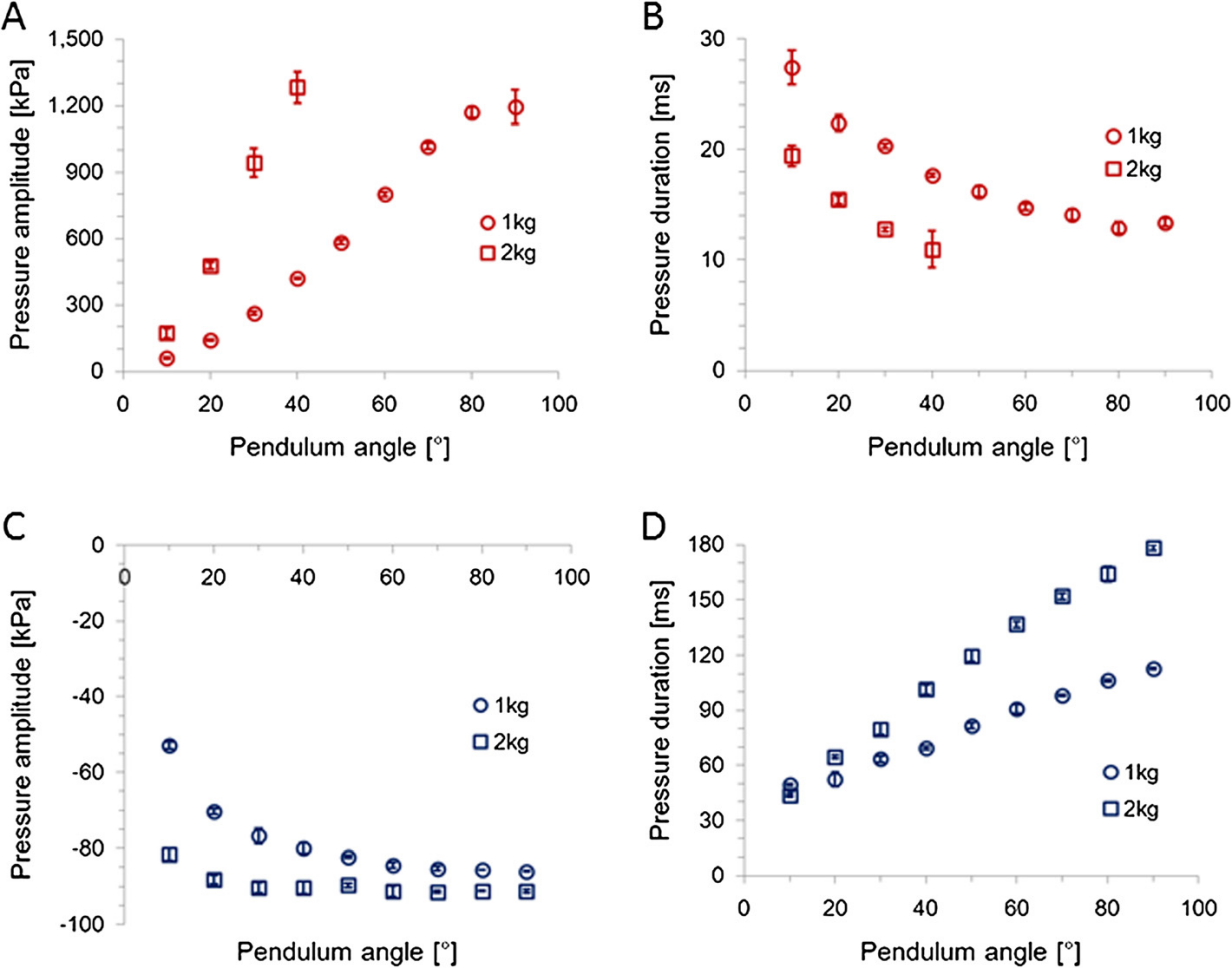
**Relation between pendulum angle and pressure amplitude or pressure duration in positive and negative pressure.** Open circles and open squares indicates pressures generated using a 1 and a 2 kg weight struck with a pendulum, respectively. Panel **A** is the change in amplitude of positive pressure. Panel **B** is the change in duration of positive pressure. Panel **C** is the change in amplitude of negative pressure. Panel **D** is the change in duration of negative pressure. Results are expressed as the mean ± standard deviation of three independent experiments.

**Figure 3:**
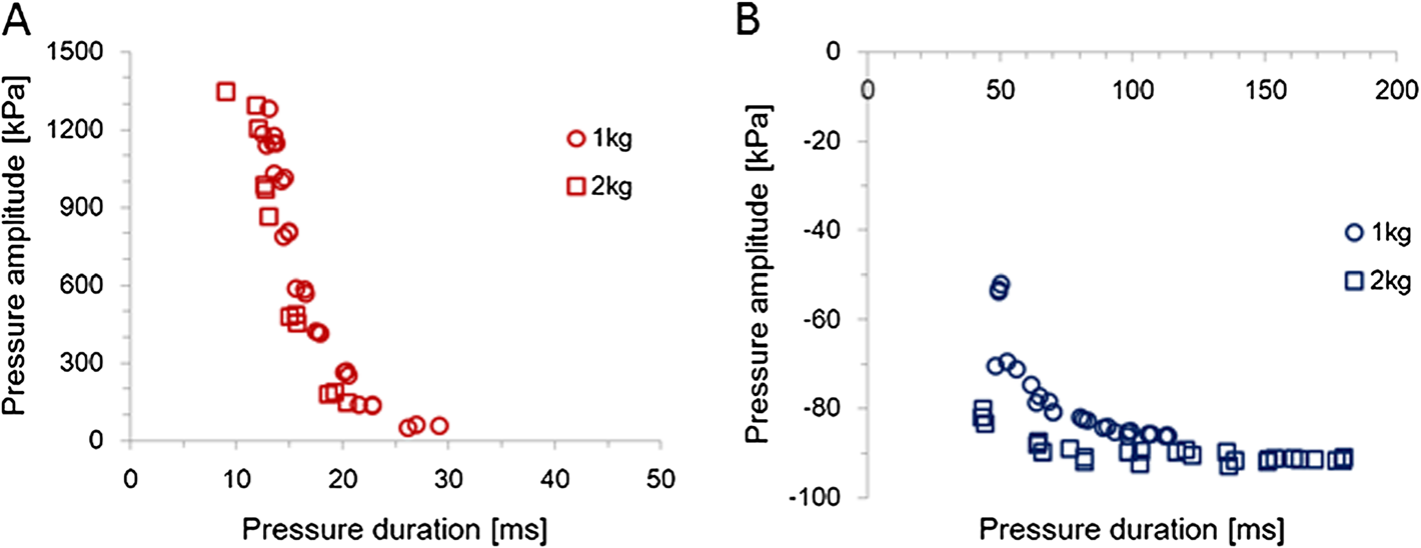
**Combination of amplitude and duration of positive pressure and negative pressure.** Open circles and open squares indicate pressures generated using a 1 and a 2 kg weight struck with a pendulum, respectively. The amplitudes are 53–1348 kPa and the durations are 9–29.1 ms in positive pressure **(A)**. The amplitudes are −52–−93 kPa and the durations are 42.9–179.5 ms in negative pressure **(B)**.

The amplitude and duration of the generated pressure were reproducible results and were controlled by the incidence angle of the pendulum and the magnitude of the weight. The amplitude of positive and negative pressures increased with the angle and weight (Figure 2A and C); however, the change in negative pressure was smaller than that in positive pressure. The duration of positive pressure decreased with increasing angle and weight (Figure 2B), whereas that of negative pressure increased with increasing angle and weight (Figure 2D); the change in negative pressure was larger than that in positive pressure. The amplitude and duration of the positive pressure were 53–1348 kPa and 9–29.1 ms, respectively (Figure 3A). The amplitude and duration of the negative pressure were −52–−93 kPa and 42.9–179.5 ms, respectively (Figure 3B).

### Disruption of capillary-like structure

**Figure 4:**
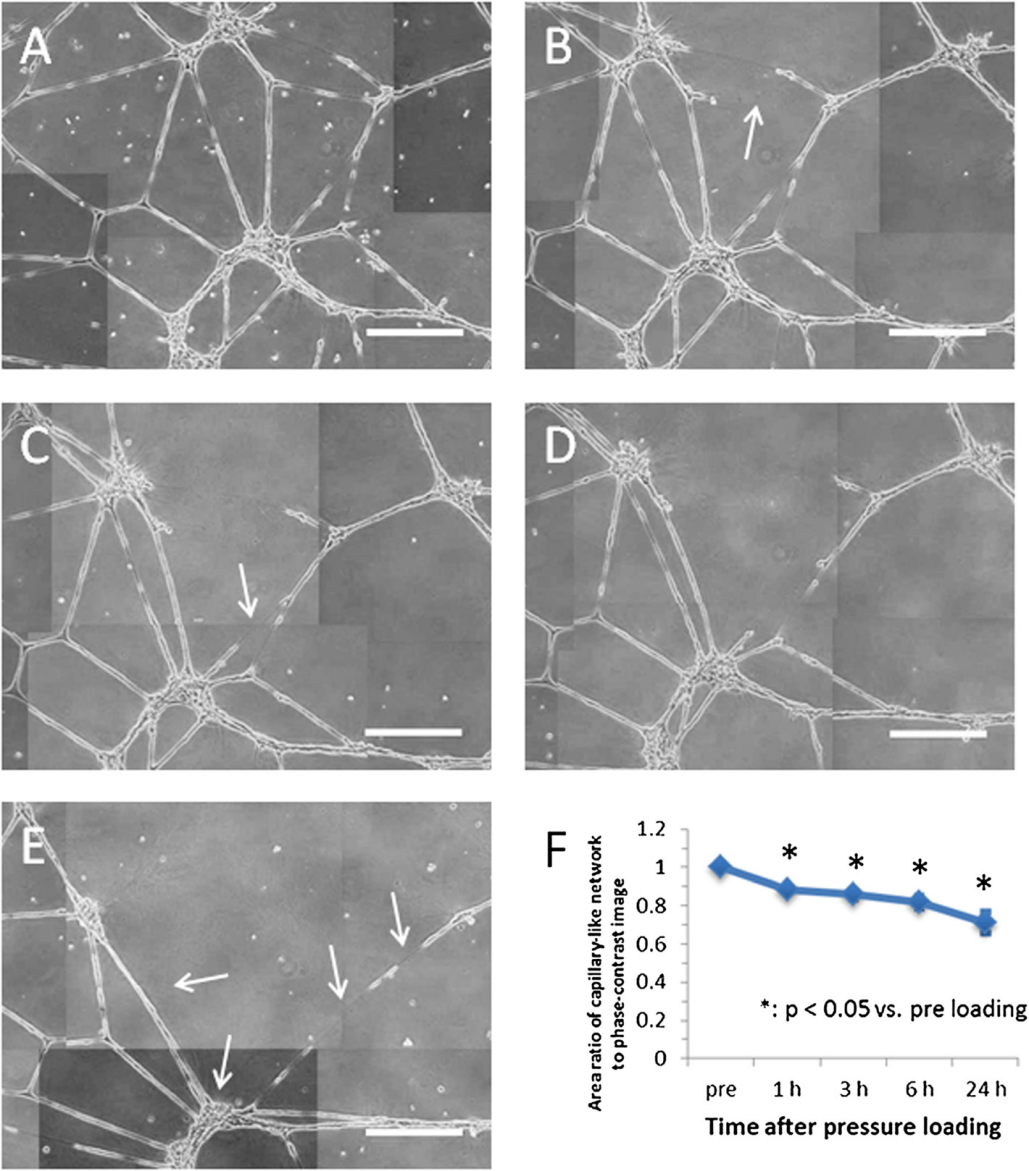
**Phase-contrast images and density of capillary-like network formed by endothelial cells after loading of impulsive pressure.** Endothelial tube formations were observed before loading **(A)** and at 1 h **(B)**, 3 h **(C)**, 6 h **(D)**, and 24 h **(E)** after loading of impulsive pressure with amplitude of 1 MPa. Arrows indicate the disruption of the capillary-like structure. Scale bar is 400 μm. The area ratio of the capillary-like network to phase-contrast images was calculated time-dependently **(F)**.

The changes in the capillary-like network formed by endothelial cells were observed using an inverted microscope before loading and at 1, 3, 6, and 24 h after loading of the impulsive pressure with amplitude of −90–1200 kPa. The capillary-like structure was broken down after 1 h following impulsive pressure, and the capillary network gradually disappeared until 24 h after loading (Figure 4). The disappearance of the capillary network was not observed at a pressure amplitude of 350 kPa or less (data not shown). Therefore, a pressure amplitude up to 350 kPa was employed to evaluate the endothelial cell function, which serves as a measurement of TEER.

### Decrease in TEER associated with changes in VE-cadherin localization

**Figure 5:**
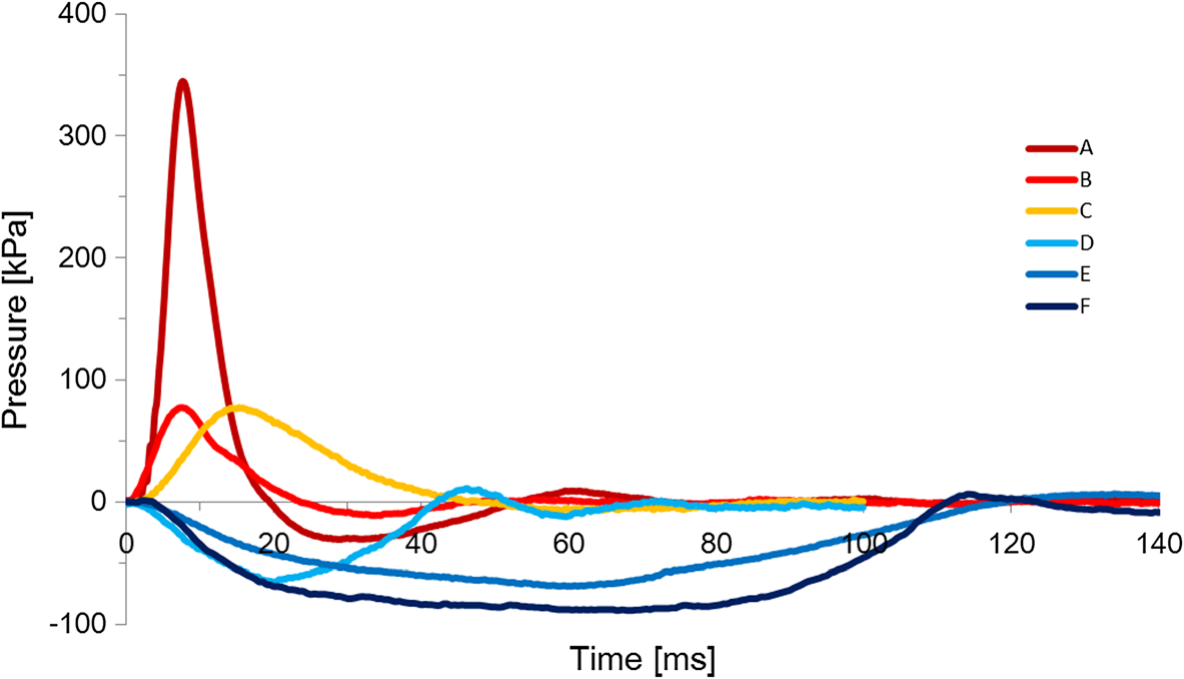
**Change in transendothelial electrical resistance (TEER) after loading of impulsive pressure.** The TEER before loading of impulsive pressure is 1.0. The * symbol represents a statistically significant difference (p < 0.05) versus pre-loading TEER at each condition using Steel’s multiple comparison test. Results are expressed as the mean ± standard error of 3–6 independent experiments.

**Figure 6:**
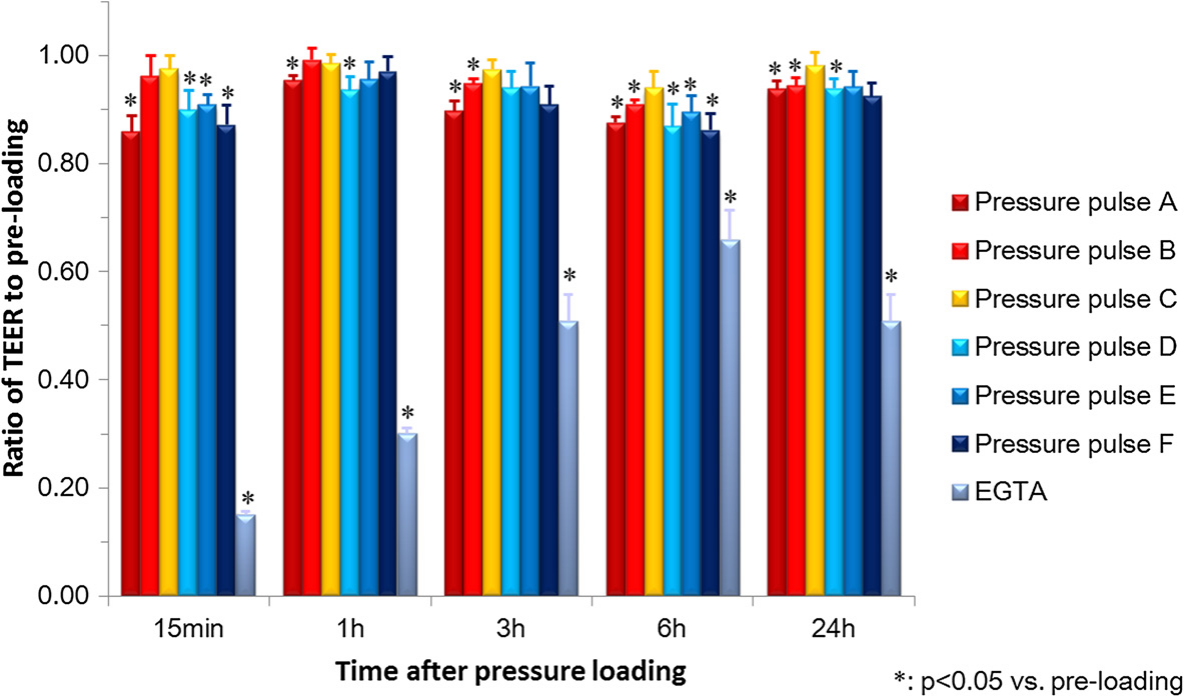
**Representative waveform of pressure pulses applied to endothelial cells.** A pressure pulse with a maximum pressure and duration of 350 kPa and 20 ms **(A)**, 70 kPa and 20 ms **(B)**, 70 kPa and 40 ms **(C)**, −70 kPa and 40 ms **(D)**, −70 kPa and 100 ms **(E)**, and −90 kPa and 100 ms **(F)**, respectively.

**Figure 7:**
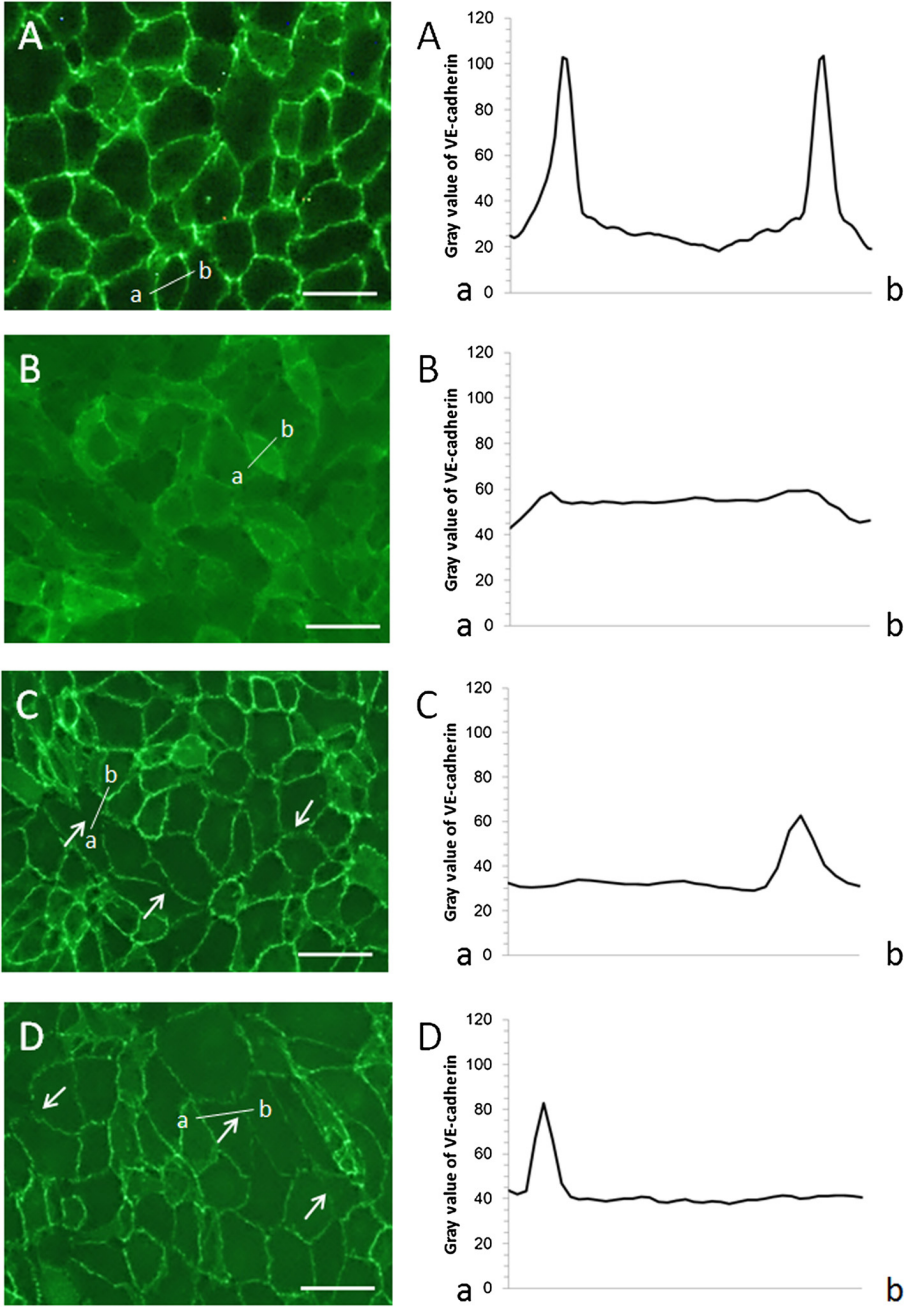
**Fluorescent images and grey value profiles of VE-cadherin in endothelial cells after loading of impulsive pressure.** VE-cadherin was observed in endothelial cells in a static culture **(A)**, incubated with EGTA **(B)**, and subjected to pressure pulses A **(C)** and F **(D)**. Scale bar is 50 μm. Arrows indicate the loss of VE-cadherin in cell-cell contacts. The grey values of VE-cadherin were plotted along the cross-sectional surface of an endothelial cell along line a–b. The grey value of VE-cadherin increased in continuous cell-cell contacts in static culture **(A)**, but remained relatively constant throughout the cells after EGTA incubation **(B)**. The grey value did not change with the loss of VE-cadherin in cell-cell contacts after pressure loading (**C** and **D**).

HUVEC were subjected to six types of impulsive pressure, and the changes in TEER were measured before loading and at 15 min, 1 h, 3 h, 6 h, and 24 h after loading (Figure 5). The representative waveforms and amplitudes and durations of the applied pressure pulses are shown in Figure 6 and Table 1, respectively. The TEER after loading of pressure pulse A with an average amplitude of 352 kPa and average duration of 23 ms significantly decreased at all time points compared to the TEER of pre-loading, and it reached ~85% at 15 min and 6 h after loading. The TEER after loading of pressure pulse B with an average amplitude of 73 kPa and average duration of 27 ms significantly decreased after 3 h post-loading compared to the TEER of pre-loading, and it reached ~90% at 6 h after loading. The TEER after loading of pressure pulse C with an average amplitude of 70 kPa and average duration of 44 ms did not change significantly at all time points compared to the TEER of pre-loading. The TEER after loading of pressure pulse D with an average amplitude of −72 kPa and average duration of 41 ms significantly decreased except at 3 h post-loading compared to the TEER of pre-loading, and it reached ~90% at 15 min and 6 h after loading. The TEER after loading of pressure pulse E with an average amplitude of −67 kPa and average duration of 104 ms significantly decreased to approximately 90% at 15 min and 6 h after loading compared to the TEER of pre-loading. The TEER after loading of pressure pulse F with an average amplitude of −91 kPa and average duration of 108 ms significantly decreased to approximately 85% at 15 min and at 6 h after loading compared to the TEER of pre-loading. On the other hands, the TEER after exposure to EGTA largely decreased to approximately 15% at 15 min and then gradually recovered to approximately 50% at 24 h after loading. Additionally, VE-cadherin localization after loading six types of impulsive pressure and after exposure to EGTA was observed using a fluorescence microscope. VE-cadherin was localized along the cell-cell contacts continuously in the static culture (Figure 7A) and throughout the cells after EGTA incubation (Figure 7B). In contrast, VE-cadherin was observed intermittently along the cell-cell contacts and weakly in the cytoplasm after the loading of pressure pulses A and F, which induced the largest decrease in the TEER of six types of impulsive pressure (Figure 7C and D). Changes were rarely observed in VE-cadherin localization in endothelial cells subjected to pressure pulses except A and F, shown in Table 1.

**Table 1:** Amplitude and duration of pressure pulses applied to endothelial cells

**Pressure pulse**	**Amplitude [kPa]**	**Duration [ms]**
A	352 ± 11	23 ± 2
B	73 ± 6	27 ± 1
C	70 ± 7	44 ± 0
D	−72 ± 7	41 ± 1
E	−67 ± 1	104 ± 4
F	−91 ± 3	108 ± 4

## Discussion

The positive pressure in this study was comparable to that of other fluid percussion models and cadaver experiments, and the positive pressure waveform included a small negative pressure. For example, a negative pressure with a peak amplitude of −25 kPa followed a positive pressure with a peak amplitude of 350 kPa (pressure pulse A shown in Figure 6). The secondary component of the negative pressure in the positive pressure waveform could not be suppressed in our pressure loading device; however, the complex waveforms of pressure fluctuation were measured in cadaver experiments [7]. Therefore, it would be necessary to control the pressure fluctuation generated by this device and to investigate the effect of secondary or multiple pressure components. The negative pressure, too, was comparable in terms of amplitude, but the duration was much longer. The pendulum impactor employed by Shepard et al. [10] generated a transient pressure pulse with an amplitude of 122–578 kPa and duration of 20–30 ms. The weight-dropping impactor employed by Orfeo et al. [11] generated a transient pressure pulse with an amplitude of 122–1013 kPa and duration of 2–5 ms. Hardy et al. [7] reported that a positive pressure with an average peak amplitude of 51.0 ± 32.1 kPa and average duration of 20.4 ± 20.4 ms and negative pressure with an average peak amplitude of −55.3 ± 36.7 kPa and average duration of 4.7 ± 2.5 ms were measured at the coup and contrecoup locations in 31 impacts using 7 human cadaver head specimens. In our pressure loading device, the extended duration of negative pressure correlated with the mechanical energy from the pendulum (Figure 2D). The duration of negative pressure increased because mechanical energy was not used to increase the peak amplitude but to extend the duration, because hydrostatic fluid pressure in the cylinder was not below −100 kPa even when the compressive stress of the piston, i.e. mechanical energy from the pendulum, increases with the angle and weight, unlike the positive pressure. Therefore, a piston with smaller energy could be used; however, it would be difficult to produce a negative pressure with a shorter duration equivalent to a real-world head impact.

This study demonstrated that impulsive pressure correlated with the disruption of capillary-like structures over time after impact. Recently, some studies have suggested that delayed and progressive haemorrhage during the first several hours after head impact are attributed to molecular events initiated at the time of impact, which lead to later structural failure of microvessels [18]. Simard et al. showed that necrotic death of endothelial cells results in physical disruption of capillaries, leading to the extravasation of blood and formation of petechial haemorrhage in a contusive animal experiment [19]. However, the mechanical factor that influences the molecular abnormality has not been identified. A further detailed investigation of the molecular mechanism of capillary fragmentation using an *in vitro* model is required.

Several previous studies have reported that mechanical stimuli regulate endothelial barrier function. A laminar fluid shear stress increases TEER transiently [20-22]; however, a disturbed flow decreases TEER [23]. Physiological cyclic stretch suppresses the decrease in TEER induced by thrombin, a barrier-disruptive chemical agonist, although a pathological cyclic stretch enhances the decrease [24,25]. Hydrostatic pressure below 16 mmHg (~2 kPa) continuously decreases the TEER in human Schlemm’s canal and Madin-Darby canine kidney cell monolayers during 30 min [26]. We also demonstrated that impulsive pressure decreased the TEER in HUVEC, although the decrease in TEER by chemical stimuli such as EGTA was larger. In addition, a pressure pulse with larger amplitude decreased the TEER more if the duration was similar. For example, pressure pulse A of 352 kPa decreased TEER more than pressure pulse B of 73 kPa, and pressure pulse F of −91 kPa decreased TEER more than pressure pulse E of −67 kPa. A pressure pulse with shorter duration decreased TEER more if the amplitude was similar. For example, pressure pulse B of 27 ms decreased TEER more than pressure pulse C of 44 ms, and pressure pulse D of 41 ms decreased TEER more than pressure pulse E of 104 ms. A negative pressure decreased TEER more than did a positive pressure if the amplitude and duration were similar, such as pressure pulses C and D. The increase in the peak amplitude, duration, or impulse of the applied positive pressure correlated strongly with the decrease in TEER after 15 min post-injury, with a correlation coefficient of −0.75, 0.54, or −0.66, respectively. The correlation coefficients for negative pressure were 0.32, −0.10, and 0.27, respectively. Gurdjian et al. reported that a shorter duration and higher amplitude correlated with the degree of cerebral concussion in the experimental concussion of dogs [4]. Hue et al. [27] reported that the blast overpressure acutely reduced the TEER in endothelial monolayers from a mouse brain microvascular cell line in a dose-dependent manner and that the decrease in TEER was strongly correlated with the fluid peak overpressure or fluid impulse, as opposed to fluid duration. These reports corroborate our results, although insufficient combinations of peak amplitude and applied pressure durations have been used in this study. However, in this study, the TEER decreased when the pressure duration decreased against the increase in fluid duration. These differences in positive and negative correlation might be due to differences in impulsive pressure and blast overpressure. Moreover, we demonstrated that the decrease in TEER, i.e. the increase in endothelial permeability, is larger immediately and at 6 h after pressure loading compared to other measurement times. Cernak et al. reported that BBB permeability to Evans blue dye in the cortex and hippocampus of rats increased at 20 min and 4 h following moderate diffuse traumatic brain injury [28]. These results suggested that the amplitude and duration of the impulsive pressure generated by head impact and the elapsed time after head impact influence the degree of increase in endothelial permeability leading to the brain injury.

Endothelial cells, which cover the intravascular lumen in the monolayer, adhere to neighbouring endothelial cells; these endothelial cell-cell adhesions mainly consist of adherens junctions (AJs) and tight junctions (TJs). The endothelial permeability is regulated in part by the dynamic opening and closing of AJs, which are largely composed of VE-cadherin [17]. EGTA treatment caused TEER to drop to 30% of the initial value associated with VE-cadherin localized throughout the cells, as shown in Figure 7B, in this study [16]. VE-cadherin monoclonal antibodies lead to an increase in vascular permeability in the heart and lungs of adult mice [29] and induce the redistribution of VE-cadherin from intercellular junctions to the cell membrane, as shown in Figure 7B, and block endothelial tube formation in cultured HUVEC [30]. In this study, the exposure to EGTA reduced TEER to 15% at 15 min associated with VE-cadherin localized throughout the cells. Therefore, this complete disruption of the integrity of endothelial cell-cell junctions could induce structural failure of the capillary, leading to bleeding such as cerebral contusion. On the other hand, the loading of impulsive pressure reduced TEER to 86–97% at 15 min associated with VE-cadherin localized intermittently along cell-cell contacts and partially in the cytoplasm. Therefore, this weak disruption of the integrity of endothelial cell-cell junctions could induce the opening of the BBB without structural failure of the capillary, leading to an increase in vascular permeability such as vasogenic brain edema.

On the other hand, TJs including occludin and claudins also regulate the paracellular permeability of endothelial cells [31]. The loss of occludin and zonula occludens-1 (ZO-1), which is a submembranous tight junction-associated protein, opens the BBB in the cerebral vascular endothelium of rats [32]. Blast overpressure reduces ZO-1 in mouse brain microvascular endothelial cells associated with an increase in hydraulic conductivity and decrease in TEER [27]. However, HUVEC shows lower TEER and higher permeability to FITC-dextran than human brain microvascular endothelial cells (HBMEC) owing to a lack of occludin expression and lower expression of ZO-1 [33]. Therefore, further studies using HBMEC are required to elucidate the influence of impulsive pressure generated by head impact on BBB breakdown.

## Conclusions

We developed a pressure loading device that can generate a positive pressure with amplitude of 53–1348 kPa and duration of 9–29.1 ms and a negative pressure with amplitude of −52–−93 kPa and duration of 42.9–179.5 ms reproducibly by controlling the angle and weight of a pendulum. We used this device to demonstrate that impulsive pressure broke down the endothelial tube formation and decreased the TEER, which is associated with the change in VE-cadherin localization. In addition, the degree of decrease in TEER was larger when loading an impulsive negative pressure with larger amplitude and shorter duration. These results suggest that the transient changes in intracranial pressure during head impact would impair endothelial tube structures like capillaries and endothelial permeability by the disruption of the integrity of endothelial cell-cell junctions. The degree of increase in endothelial permeability depends on the amplitude, duration, and direction (compressive and tensile) of the impulsive pressure.

## Competing interests

The authors declare that they have no competing interests.

## Authors’ contributions

HN designed the study and performed the statistical analysis. KI measured the device outputs and the TEER. SA observed the immunofluorescence staining. AK participated in the sequence alignment. SA conceived the study, participated in its design and coordination, and helped to draft the manuscript. All authors have read and approved the final manuscript.
